# Scientific Items

**Published:** 1882-02-20

**Authors:** 


### SCIENTIFIC ITEMS.

**The Musquito as a Carrier of Disease.**—A correspondent inquires whether there is "anything in the newspaper statement that musquitoes are the agents for introducing dangerous parasites into the human blood." We are pained to be obliged to say that there is good ground for this addition to the disreputable "record" of the insect. The discovery was made a year or more ago—we cannot give the exact date—and has since been fully confirmed by further investigation. Dr. Meisoner of Leipsic, in a German medical magazine, has lately summed up what is known of the parasitic infection of the blood, and the following is an abstract from what he says of the *filiaria sanguinis hominis*:—This parasite has been very thoroughly studied by Manson, of Amoy, China, and Bancroft of Brisbane, Australia. The filiaria, while it may at times be present in the blood without giving rise to any symptoms, at other times appears beyond question to be the cause of chyluria, elephantiasis, etc. The mode of its action would seem purely mechanical. The parasite in the blood or lymph channels and its accumulation at a given point gives rise to lymphorrhagia or inflammation. Two curious facts have recently come to light regarding the parasite. One is that the musquito acts as a carrier; sucking the filaria with the blood of an affected person, it afterwards deposits the ova or embryos, which have meantime hatched, in the water when it lays its own eggs. These embryos are then swallowed in the drinking-water by another victim; and so the circle of disease is completed. Another and a very curious fact regarding the filaria was lately discovered; this is that it is a nocturnal parasite. During the day the filiariæ lie dormant at some point in the victim's circulation, but at night they sally forth and rove the currents of the blood the night long.—[Boston Journal of Chemistry.

**Telephone Sound of Voices.**—It is noticed that the telephone gives a particular accent to the peculiarities of voice. A growling voice is surlier, a sharp voice more sharp; like the photograph, it makes matters worse. There is nothing like the sun picture for bringing out the hidden peculiarities of a face. They may be hidden in real life, in trained expressions, or favor of complexion, but the photograph is sure to seize them, almost to caricature. So the telephone, too, has a trick of making the voice "a little more so" than it is, in whatever direction its defects lie. This playfulness of the telephone might stand in its way sometimes, if it should come generally into use for critical purposes; but does not appear to have been in Briggsville, Mass., where a croupy child was brought to cough into a telephone so that the doctor, some miles away, at North Adams, could hear and prescribe for it It probably made the case out at least as bad as it could for the little sufferer, who was speedily relieved by the return prescription over the wire.—[Ex.

**Ancient Division of Time.**—The German Heusler has suggested on the same point that the ancients did not divide time as we do. Previous to the age of Abraham the year, among some people of the East, was only three months, or a season; so that they had a year of spring, one of summer, one of fall, and one of winter. The year was extended so as to consist of eight months after Abraham, and of twelve months after Joseph. Voltaire rejected the longevity assigned to the patriarch of the Bible, but accepted without question the stories of the great age attained by some men in India, where, he says, "It is not rare to see old men of one hunered and twenty years." The eminent French physiologist, Flourens, fixing the complete development of man at twenty years, teaches that he should live five times as long as it takes him to become an adult. According to this author the moment of a complete development may be recognized by the fact of the junction of the bones with their apophyses. The junction takes place in horses at five years, and the horse does not live beyond twenty-five years; with the ox, at four years, and it does not live over twenty years; with the cat at eighteen months, and that animal rarely lives over ten years. With man, it is effected at twenty years, and he only exceptionally lives beyond one hundred years. The same physiologist admits, however, that human life may be exceptionally prolonged under certain conditions of comfort, sobriety, freedom from care, regularity of habits, and observance of the rules of hygiene; and he terminates his interesting study of the last point ("De la Longevite humaine") with the aphorism, "Man kills himself rather than dies."

**The Dentaphone.**—In answer to question No. 275 regarding the "Dentaphone" will say: I have used one for about 18 months. Am quite deaf and have false teeth; yet with the dentaphone I hear quite well; in fact can do business nearly as well as ever. It does not work on all alike, however. In some the nerves from the teeth or gums are paralyzed, so that no sound is conveyed. If the nerves are perfect, any one will be greatly benefitted after one or two weeks practice. To tell whether the nerves are alive, take a small stick a foot long; place one end on the sounding board of a piano, the other against the teeth. If it sounds louder on striking the piano keys, you can learn to use the dentaphone.—W. W. L., Kansas City, Mo.—[Drug Cir.

**The Pennsylvania Railroad** has in use an automatic track tester which discovers faults in the track not ordinarily appreciable to the eye, and makes a record of them which indicates their precise locality, and all this while the machine is passing over the road at from fifteen to twenty-five miles an hour. It has the external appearance of a baggage car, but inside is fitted up with self-registering apparatus, electric clock, etc. A bad joint between the rails registers itself by the jolt it causes to the delicately hung car. Errors of level in the track are recorded by pencils on ruled paper, and so nicely arranged that the variations of an eighth of an inch are made manifest.—[Mechanical News.

